# PXR and 4β-Hydroxycholesterol Axis and the Components of Metabolic Syndrome

**DOI:** 10.3390/cells9112445

**Published:** 2020-11-09

**Authors:** Janne Hukkanen, Jukka Hakkola

**Affiliations:** 1 Research Unit of Internal Medicine, Biocenter Oulu, Medical Research Center Oulu, University of Oulu and Oulu University Hospital, POB 5000, FI-90014 Oulu, Finland; 2 Research Unit of Biomedicine, Biocenter Oulu, Medical Research Center Oulu, University of Oulu and Oulu University Hospital, POB 5000, FI-90014 Oulu, Finland

**Keywords:** pregnane X receptor, 4β-hydroxycholesterol, metabolic syndrome, hypertension, reverse cholesterol transport, hyperglycemia, obesity

## Abstract

Pregnane X receptor (PXR) activation has been found to regulate glucose and lipid metabolism and affect obesity in response to high-fat diets. PXR also modulates vascular tone. In fact, PXR appears to regulate multiple components of metabolic syndrome. In most cases, the effect of PXR action is harmful to metabolic health, and PXR can be hypothesized to play an important role in metabolic disruption elicited by exposure to endocrine-disrupting chemicals. The majority of the data on the effects of PXR activation on metabolic health come from animal and cell culture experiments. However, randomized, placebo-controlled, human trials indicate that the treatment with PXR ligands impairs glucose tolerance and increases 24-h blood pressure and heart rate. In addition, plasma 4β-hydroxycholesterol (4βHC), formed under the control of PXR in the liver, is associated with lower blood pressure in healthy volunteers. Furthermore, 4βHC regulates cholesterol transporters in peripheral tissues and may activate the beneficial reverse HDL cholesterol transport. In this review, we discuss the current knowledge on the role of PXR and the PXR–4βHC axis in the regulation of components of metabolic syndrome.

## 1. Introduction

Metabolic syndrome (MetS) is a cluster of risk factors that increases the risk of coronary heart disease, type 2 diabetes, and stroke [1,2]. Its prevalence has increased significantly in recent decades, with up to one third of the adult population having MetS [3,4]. The components of MetS include abdominal obesity, hyperglycemia, high blood pressure, low HDL cholesterol, and elevated triglycerides. MetS can be diagnosed if three or more of the following findings are present: a waist circumference of ≥94 cm in men and ≥80 cm in females, elevated triglyceride levels (≥1.7 mmol/L), low levels of HDL cholesterol (<1.0 mM in men, <1.3 mmol/L in women), high blood pressure (≥130/ ≥85 mmHg), and increased fasting glucose (≥5.6 mmol/L) [5]. Drug treatment for hypertension, low HDL cholesterol, hypertriglyceridemia, or hyperglycemia are included as alternate indicators.

The rising prevalence of MetS is largely attributed to the sedentary lifestyle and the obesity epidemic afflicting the global population. However, exposure to environmental pollutants has been suggested to be an additional risk factor for the development of MetS [6,7]. These metabolism-disrupting or endocrine-disrupting chemicals (EDCs) are especially linked to the incidence of type 2 diabetes and its preceding phenomena, namely insulin resistance and prediabetes [8,9]. In addition, a closely related environmental obesogen hypothesis has been proposed suggesting a causative role for EDCs in the pathogenesis of obesity [10,11]. There are also links between exposure to EDCs and the other components of MetS, i.e., hypertension, low HDL cholesterol (HDL-C), and elevated triglycerides [7]. 

Several molecular mechanisms mediating the effects of EDCs on metabolism have been proposed, including estrogen and other sex hormone receptors, glucocorticoid receptor, peroxisome proliferator-activated receptors, and xenobiotic-sensing receptors such as aryl hydrocarbon receptor (AhR), constitutive androstane receptor (CAR), and pregnane X receptor (PXR) [6,12]. In this review, we concentrate on the effects of PXR on MetS and discuss the possibility that, due to its roles as a xenosensor and a regulator of glucose and lipid metabolism as well as blood pressure [13,14], PXR activation could contribute to the development of MetS.

## 2. PXR and Obesity

Obesity is a major driver of MetS. The main cause of obesity is thought to be overnutrition, i.e., prolonged positive energy balance. Furthermore, certain chemicals have been shown to promote obesity. These so-called obesogens are considered a subclass of EDCs [11,15]. As a major xenosensor and a regulator of energy metabolism [13], PXR represents a putative target for the EDCs to mediate their weight-gain-promoting and other metabolic effects [16].

PXR has been reported to modulate the development of obesity in response to high-fat diets (HFDs). Several studies have observed reduced weight gain in *Pxr*-knockout (KO) mice under HFD, suggesting that PXR deficiency could protect against diet-induced obesity [17,18,19]. The *Pxr* KO did not affect the food intake [17,18], suggesting that energy expenditure must be altered. Indeed, He et al. observed increased oxygen consumption in *Pxr*-KO mice [18]. Furthermore, a recent study found increased fibroblast growth factor 15 (FGF15) expression, reduced serum bile acids level, and increased fecal lipid content in the HFD-fed *Pxr*-KO mice [19]. These findings suggest that *Pxr* KO reduces lipid absorption, possibly through FGF15-mediated regulation of bile acid synthesis [19].

While the results in the *Pxr*-KO models suggest that PXR plays an obesity-promoting role, there are also contradictory results. Ma and Liu reported that treatment of obesity-prone ARK/J mice with PXR ligand pregnenolone 16α-carbonitrile (PCN) inhibited weight gain under HFD and at the final time point of seven weeks, the weight of the PCN-treated mice was similar to the chow-fed mice [20]. The prevention of HFD-induced weight gain by PCN was due to reduced fat mass [20]. The PCN-treated mice ate less; however, the caloric intake ratio to the bodyweight was actually slightly increased [20]. The authors observed higher body temperature in the PCN-treated mice upon cold exposure and induction of thermogenesis genes in the brown adipose tissue, suggesting enhanced thermogenesis. Thus, although the oxygen consumption was not measured, these results suggest that the brown adipose tissue activity in response to PCN could cause increased energy expenditure. There was no *Pxr*-KO control in these experiments, and therefore some uncertainty about the molecular mediator remains; however, the majority of the PCN effects are mediated by PXR [20]. Together, these results appear to suggest a puzzling conclusion that both *Pxr* KO and PXR activation attenuate HFD-induced obesity. Clearly, more studies are still needed to fully characterize the effect of PXR on diet-induced obesity in mice.

The situation is even more complex when the *PXR*-humanized mouse model is studied. In males, *PXR* humanization inhibited HFD-induced weight gain [17]. In contrast, in females, the *PXR* humanization aggravated HFD-induced obesity [21]. Although these models have humanized *PXR*, it needs to be kept in mind that otherwise they still represent mouse physiology, and thus the results cannot be directly translated to humans. No data from controlled clinical studies exist on this subject. The current view of the PXR effect on obesity has been collected in Table 1.

## 3. PXR and Glucose Homeostasis

Among the components of MetS, disruption of glucose metabolism is the one with the strongest evidence of being targeted by PXR activation. The harmful effect of PXR ligands on postprandial glucose metabolism has been established in two clinical intervention trials [22,23]. We performed a randomized, single-blind, placebo-controlled, crossover trial to investigate the effect of rifampicin, a well-established PXR agonist, on oral glucose tolerance [22]. Remarkably, relatively short, 7-day treatment with rifampicin impaired glucose tolerance as measured with oral glucose tolerance test, suggesting that PXR activation induces postprandial hyperglycemia. An analogous effect has been observed in rats with a structurally different, nonantibiotic rodent PXR ligand PCN, supporting the idea that PXR indeed mediates the effect [22,24]. Furthermore, four-day treatment of mice with PCN similarly impaired glucose tolerance [25]. The effect was abolished by *Pxr* KO, confirming the involvement of PXR. Studies in rats and humans indicated that the disruption of glucose tolerance by PXR agonists is not mediated by incretin hormones such as glucagon-like peptide-1 [26].

Stage et al. performed a clinical trial using 21-day treatment with St. John’s wort, an herbal medicine with established PXR-activation property [23]. Comparison of the oral glucose tolerance test results before and after the treatment indicated that St. John’s wort caused impairment of glucose tolerance. Strikingly, the impairment remained six weeks after cessation of the treatment, suggesting a long-lasting effect. However, the design of that study was not ideal as there was no control arm.

**Figure 1:**
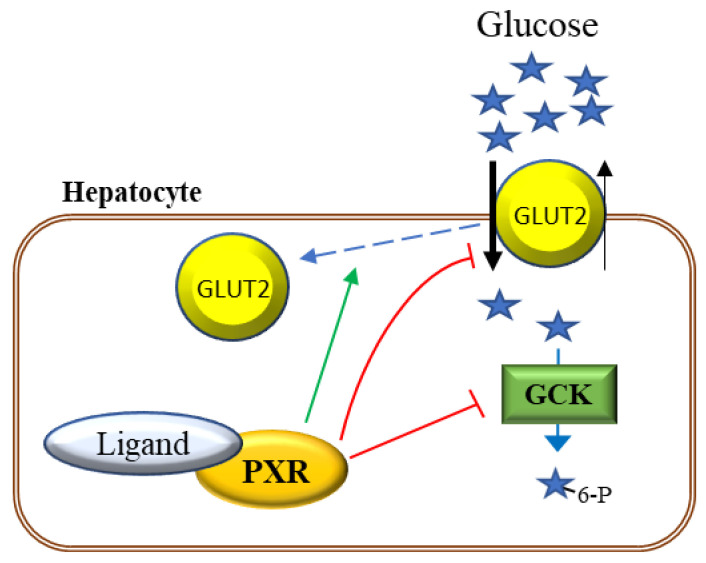
Mechanisms mediating the effect of PXR activation on hepatic glucose uptake. In the postprandial state, GLUT2 glucose transporter facilitates glucose uptake into the hepatocytes. In the next step of glucose utilization, glucose is phosphorylated by GCK. Ligand-activated PXR represses expression of both GLUT2 and GCK. PXR activation also relocates GLUT2 from the plasma membrane to the cytosol. Together, these alterations reduce postprandial hepatic glucose uptake.

The mechanisms involved in the PXR-elicited glucose intolerance have been characterized in some detail and appear to be due to suppression of hepatic glucose uptake [25]. At the molecular level, PXR represses glucose transporter 2 (GLUT2) mRNA and protein expression in liver, both constitutively and in response to glucose [22,25]. Furthermore, immunohistochemistry of the PCN-treated and control mice livers suggested that the PXR activation promotes GLUT2 internalization from the plasma membrane to the cytosol [25]. Dysregulation of GLUT2 function may play an important role in the reduced hepatic glucose uptake. Additionally, the next step in the glucose uptake, phosphorylation by glucokinase (GCK), may also be repressed since the GCK mRNA expression tended to be downregulated in the mouse and rat liver by PXR activation, although the effect was not as consistent as that of the GLUT2 repression [22,25]. In HepG2 cells, PXR ligands rifampicin and atorvastatin, unlike the non-PXR-ligand pravastatin, repressed GLUT2 and GCK protein levels [24]. Knockdown of PXR had an opposite effect [24], supporting the in vivo results in rodents. Both *Glut2* and *Gck* KO mice have been shown to manifest mild hyperglycemia in a fed state, fitting with the theory that repression of these proteins involved in glucose utilization could result in postprandial hyperglycemia [27,28]. The current model of PXR’s effect on hepatic glucose uptake is presented in Figure 1.

In addition to the glucose uptake, PXR activation may affect gluconeogenesis. However, the effect may be species-specific. It is rather well established that PXR ligands repress the key gluconeogenic genes glucose-6-phosphatase (*G6Pase*) and phosphoenolpyruvate carboxykinase (*Pepck*) in mouse liver and mouse primary hepatocytes (for review, see [13] and the references therein). Mechanistically, this involves PXR-mediated transrepression through interaction with several key transcription factors regulating gluconeogenesis, including Forkhead box protein O1 (FOXO1) and cAMP response element-binding protein (CREB), or, in the case of hepatocyte nuclear factor 4 (HNF4), competition for the common coactivator PGC-1α (peroxisome proliferator-activated receptor gamma (PPARγ) coactivator 1α) [29,30,31]. 

Repression of the gluconeogenic genes is expected to result in the downregulation of gluconeogenesis and hepatic glucose output, i.e., beneficial effect in insulin resistance models. In some experimental settings, such an effect has been observed. Ma and Liu reported that PCN treatment of HFD-fed mice improved glucose tolerance [20]. As described above, this was associated with a beneficial effect on weight gain that may explain the finding. On the other hand, He et al. observed that transgenic activation of PXR with liver specific Alb-VP-*Pxr* allele worsened glucose tolerance and insulin sensitivity in the *Ob/Ob* mice [18]. In the same study, *Pxr*-KO improved glucose tolerance in the context of HFD-feeding or *Ob/Ob* genotype. Finally, Spruiell et al. reported that both *Pxr* KO and humanization aggravated HFD-induced impairment of glucose tolerance in male mice [17]. Surprisingly, in both cases, these genetic manipulations were associated with a beneficial effect on weight gain [17]. One explanation for the poor glucose tolerance in these models could have been impaired induction of GCK, possibly affecting glucose utilization [17]. At the moment, the reasons behind the different effects of *Pxr* KO on glucose tolerance observed in the studies of He et al. [18] and Spruiell et al. [17] are not clear. However, two different *Pxr* KO lines [32,33] were used in these studies, and there are reported differences between the lines, including a different effect of *Pxr* KO on basal CYP3A11 expression.

Some of the early observations on the repressive effect of PXR activation on gluconeogenesis were made in human hepatoma cell lines [29,30]. However, it has more recently been suggested that human hepatocytes may respond to PXR activation differently from mouse hepatocytes, and at least in some models, the gluconeogenic genes are induced [34,35]. The mechanism has been shown to involve serum/glucocorticoid regulated kinase 2 (SGK2). In response to pharmacological activation, demonstrated with simvastatin and rifampicin, PXR scaffolds the protein phosphatase 2C (PP2C), which dephosphorylates SGK2 [34,35]. Dephosphorylated SGK2 then acts as a coactivator for PXR in a complex binding directly or indirectly to the response elements regulating the *G6Pase* and *PEPCK* genes [35]. 

Interestingly, it has been reported that, in addition to ligand binding, VRK Serine/Threonine Kinase 1 (VRK1) mediated phosphorylation of PXR at Ser^350^ may initiate a cascade resulting in SGK2 dephosphorylation and *PEPCK* activation [36]. VRK1 was activated in low-glucose condition, and thus PXR would appear to be a part of the glucose level sensing apparatus regulating gluconeogenesis. 

It has also been reported that PXR activity is induced under high-glucose conditions and that this is related to AMPK activity; AMPK activation exhibited an inverse relation to PXR activity [37]. However, these effects were mostly demonstrated with unphysiologically high glucose concentrations. As an additional demonstration of the nutritional regulation of PXR function, the PXR-regulated transcriptome was strongly modified, both qualitatively and quantitatively, by fasting and feeding status [38]. In general, glucose feeding of the fasted mice enhanced the induction of PXR-regulated genes. However, for some genes, there was a more dramatic effect and for *Cyp8b1*, involved in bile acid synthesis, and the glucose feeding switched the response from PXR-mediated repression to induction [38].

In conclusion, PXR appears be at the crossroad of glucose and xenobiotic metabolism. There are still many inconsistencies in the data describing the effect of PXR activation (and inactivation) on glucose metabolism. and the mechanisms have been only partially characterized. However, the clinical data in humans support the notion that PXR activation is harmful to glucose metabolism and promote the development of MetS. Mechanistically, this may involve reduced hepatic glucose uptake and activation of gluconeogenesis.

## 4. PXR-4β-Hydroxycholesterol Axis and Blood Pressure Regulation

Elevated blood pressure above 130/85 mmHg is one of the key features of MetS. We had noticed in our previous trial exploring the effect of rifampicin on glucose metabolism that rifampicin appeared to increase systolic and diastolic blood pressure [22]. The office systolic blood pressure (BP) was elevated by 4.8 mmHg (*p* = 0.027), diastolic BP by 3.5 mmHg (*p* = 0.020), and heart rate by 5.0 bpm (*p* = 0.042) after 1 week of rifampicin dosing compared with the placebo arm. Therefore, in our recent study, we set out to test the hypothesis that PXR activation by rifampicin elevates 24-h BP [14]. In a randomized, single-blind, placebo-controlled, and crossover trial, 22 healthy volunteers were given rifampicin 600 mg vs. placebo daily for a week, and 24 h ambulatory BP was measured at the end of the study arms. Study personnel were blinded; subjects were not blinded because rifampicin colors urine red, rendering blinding impossible. However, volunteers were not aware of the hypothesis that BP was expected to rise. 

We showed that rifampicin elevated the mean systolic 24-hour BP by 4.7 mmHg (*p* < 0.0001) and diastolic BP by 3.0 mmHg (*p* < 0.001). The 24-h mean arterial pressure and pulse pressure were also elevated. In addition to BP indices, the mean 24 h heart rate was increased by 3.5 bpm (*p* = 0.038). Thus, we have evidence from two trials on healthy volunteers that PXR activation elevates office and 24 h ambulatory systolic and diastolic BP and heart rate [14,22].

There is no previous human study exploring the effect of PXR activation on blood pressure regulation, but a few animal studies have indicated that PXR is involved. Rifampicin treatment for 5 weeks increased plasma aldosterone in *PXR*-humanized mice [39]. Aldosterone is the adrenal mineralocorticoid regulating BP and an essential part of the renin–angiotensin–aldosterone system (RAAS). In our human study, serum renin concentration and plasma renin activity were increased by about 35%, while serum aldosterone concentration was not affected by rifampicin dosing.

In a study in spontaneously hypertensive rats and control Wistar-Kyoto rats, both BP and cytochrome P450 (CYP) 3A activity, measured as corticosterone 6β-hydroxylation, were higher in the hypertensive rats than in the control rats [40]. Crucially, BP was lowered with troleandomycin, a macrolide antibiotic and inhibitor of CYP3A. This study thus indicates that perhaps CYP3A activity and not PXR activation is the mechanism for elevated BP. 

However, PXR is expressed in human and mice aorta and mice mesenteric arteries, and direct PXR-mediated effects on the vasculature have been described [41,42,43,44]. The 2-week administration of indole 3-propionic acid, an intestinal microbiota-derived metabolite of tryptophan and a PXR agonist, reduced the endothelium-dependent vasodilation in isolated and cultured aorta in wild-type mice but not in the *Pxr*-KO mice [44]. In contrast, 7-day treatment of pregnant mice with 5β-dihydroprogesterone, a PXR agonist, enhanced endothelium-dependent relaxation of mouse mesenteric arteries in the *Pxr+/+* mice but not in the *Pxr-/-* mice [42]. The authors concluded that PXR contributes to the development of vascular adaptations to pregnancy. Thus, PXR agonists reduced vasodilatation in aorta but enhanced vascular relaxation in mesenteric arteries [42,44]. Pulakazhi Venu et al. [44] speculated that these contrasting findings could be explained by different modes of vascular tone regulation; endothelium-produced endothelial nitric oxide (NO) synthase in the aorta and endothelium-derived hyperpolarizing factors in mesenteric arteries. PXR activation is known to reduce NO release, although the model used was not linked to vascular regulation (intestinal Caco-2 cell line) [45]. It is also interesting that laminar shear stress, the atheroprotective blood flow, activates PXR in bovine aortic and human umbilical vein endothelial cells, whereas atherogenic oscillatory shear stress suppresses PXR [43]. It should also be noted that in our ambulatory BP study, the heart rate and renin activity were elevated in addition to systolic and diastolic BP [14]. All of these effects are also seen when the sympathetic nervous system is activated [46,47]. However, to the best of our knowledge, the effect of PXR activation on the sympathetic nervous system is yet to be explored.

In addition to direct PXR action on vasculature, 4β-hydroxycholesterol (4βHC), a cholesterol metabolite produced by CYP3A enzymes in the liver [48] and a liver X receptor (LXR) agonist [49], may also be linked with BP regulation. Both LXRα and LXRβ are activated by 4βHC [50]. We showed in our ambulatory BP study [14] that plasma 4βHC concentration strongly negatively correlated with 24-hour systolic BP. This negative correlation was present in both rifampicin and placebo arms (rifampicin, r = −0.69, *p* < 0.001; placebo, r = −0.70, *p* < 0.001), although rifampicin dosing elevated 4βHC concentration more than 3-fold, and 24 h BP was increased as detailed above. This could be interpreted to indicate that 4βHC is a negative regulator of BP acting to reverse PXR-mediated hypertension.

The activation of LXRα by synthetic LXR agonists T0901317 and GW3965 reduces the experimentally stimulated RAAS and/or BP [44,51,52]. However, in experimental models without prior hypertension-inducing procedures or treatments, both T0901317 and 22(R)-hydroxycholesterol treatment elevated BP in mice [53]. Both LXRα and LXRβ activate transcriptionally the expression of renin in vitro and in vivo in acute experiments [54]. In our in vitro experiments [14], 4βHC was able to activate renin expression via LXRα in human Calu-6 cells but only modestly with unphysiologically high 4βHC concentrations arguing against a direct role of 4βHC in human renin regulation. Thus, LXR activation seems to induce renin expression in rodent kidneys and a human cell model, while the experimentally stimulated RAAS activity and elevated BP are perhaps blunted by LXR activation. It is known that LXR activation induces endothelial NO synthase [55], which could possibly explain the negative association of plasma 4βHC with lower BP (circulating 4βHC–endothelial LXR activation–endothelial NO production).

In addition to CYP3A4, the polymorphic CYP3A5 enzyme synthesizes 4βHC [48]. The concentration of 4βHC increases with the number of functional CYP3A5*1 alleles [48]. A relationship between *CYP3A5* gene polymorphisms and hypertension has been suggested, but studies have yielded conflicting results [56]. There are many confounding factors such as ethnicity, the modulating effect of salt intake, and concomitant hypertension medications, some of which are CYP3A5 substrates [56]. In a meta-analysis, there was no overall effect of CYP3A5 expressor status on BP or hypertension [57]. However, in white populations, the carriers of CYP3A5*1 allele had lower systolic BP compared with noncarriers. Since the CYP3A5*1 carriers have higher circulating 4βHC concentration and higher 4βHC is associated with lower 24-hour BP [14], the increased 4βHC production could offer a mechanistic explanation for the influence of the genotype. However, more studies are still needed in this field.

To conclude, PXR activation elevates BP and heart rate in humans, while plasma 4βHC is associated with lower BP. The exact mechanisms behind these phenomena are uncertain, but direct vascular PXR action and endothelial LXR activation by 4βHC may be involved.

## 5. HDL Cholesterol Metabolism and the PXR—4βHC Axis

Low HDL-C (<1.3 mmol/L in women and <1.0 mmol/L in men) is one of the metabolic syndrome criteria. The ability of HDL to accept cholesterol from peripheral macrophages appears to be the crucial functional aspect of HDL-C metabolism and a key step in the beneficial reverse cholesterol transport (RCT) from the periphery to the liver and intestine for excretion [58,59]. Although there is no direct evidence that PXR regulates HDL-C metabolism, there are certain links between CYP3A activity and HDL-C. The treatment with PXR agonists such as carbamazepine, phenobarbital, and phenytoin is associated with increased HDL-C, while total cholesterol remains constant [60,61]. It has also been shown that CYP content of liver biopsy samples correlates with HDL-C in patients with epilepsy [62]. In psychiatric patients, CYP3A activity measured with midazolam phenotyping correlated strongly with HDL-C [63]. Phenytoin increased HDL-C and especially HDL2 but not HDL3, total cholesterol, or LDL in a placebo-controlled parallel trial in patients with low HDL-C [64]. In addition, occupational exposure to pesticide lindane, a potent PXR agonist [65], associates with remarkably high HDL-C concentrations [66].

**Figure 2:**
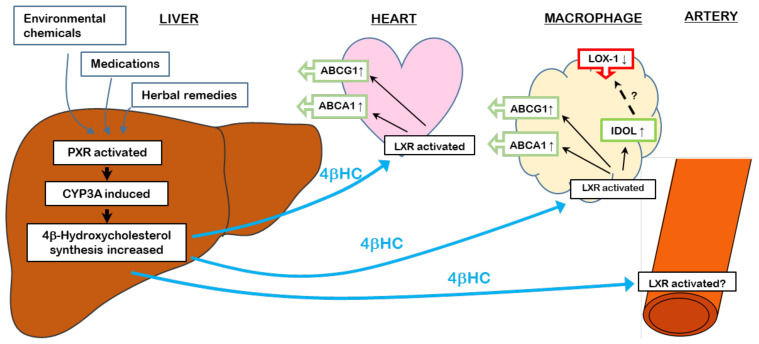
PXR–4β-hydroxycholesterol–LXR pathway as a regulator of cholesterol transporters and its putative role in vascular tone regulation. PXR activation by xenobiotics elevates circulating 4βHC, leading to the induction of cholesterol efflux transporters ABCA1 and ABCG1 and the repression of cholesterol influx transporter LOX-1. In addition to ABCA1 and ABCG1, inducible degrader of the LDL receptor (IDOL), another LXR target, is induced by 4βHC. The exact mechanism of LOX-1 repression is currently not known. The effects of 4βHC in vascular tone regulation are yet to be explored.

We have recently demonstrated that 4βHC, through its LXR agonism, could be a link between PXR activation and HDL-C metabolism as it regulates the expression of cholesterol transporters in macrophages [67]. The expression and function of efflux transporter ATP-binding cassette transporter A1 (ABCA1) and ABCG1 were induced by 4βHC in vitro. Both *ABCA1* and *ABCG1* are known transcriptional targets of LXR [58]. The efflux, facilitated by ApoAI as an acceptor for cholesterol, was augmented by 4βHC more than the efflux to HDL2 [67]. Furthermore, 4βHC incubation repressed the expression and function of influx transporter lectin-like oxidized LDL receptor-1 (LOX-1). Thus, we could show that 4βHC is able to stimulate cholesterol efflux in the macrophages, the starting point of the beneficial RCT pathway. We also showed in humans and rats that dosing with prototypical PXR agonists rifampicin and PCN was able to induce plasma/serum 4βHC significantly (>2-fold in humans and >8-fold in rats). Crucially, the expression of ABCA1 mRNA in mononuclear cells of healthy volunteers in vivo was induced by rifampicin dosing. In rats, PCN induced the expression of ABCA1 and ABCG1, especially in the heart in vivo. Thus, the evidence suggests the existence of a novel hepatic PXR–circulating 4βHC–peripheral LXR pathway that links the hepatic xenobiotic exposure and the regulation of cholesterol transport in peripheral tissues [27] (Figure 2). It may be incidental, but hepatic CYP3A4 levels, plasma 4βHC and HDL-C levels are all higher in women than men [68].

The results reviewed above indicate that PXR activation and especially elevated 4βHC levels could be beneficial factors in the regulation of HDL metabolism. This contrasts with the mostly harmful effects of PXR action on the other components MetS (obesity, hypertension, glucose metabolism). However, as obesity is a major driver of the MetS and both CYP3A4 activity and circulating 4βHC are known to be repressed by obesity and MetS [69,70], the repression of hepatic CYP3A4–circulating 4βHC–peripheral LXR pathway could link obesity and disrupted HDL-C metabolism in the obese patients with MetS.

## 6. PXR and Hypertriglyceridemia

Elevated triglyceride level is one of the diagnostic criteria for MetS. However, unlike the other components of the MetS, PXR activation appears to have little effect on the plasma triglyceride level. Efavirenz and quetiapine both induced plasma cholesterol through mechanisms dependent on PXR; however, they did not affect plasma triglyceride level [71,72]. Furthermore, the *Pxr* KO did not affect plasma triglyceride levels under HFD [17,18]. In contrast, PXR activation has been observed in many studies to promote liver steatosis. Although non-alcoholic fatty liver disease (NAFLD) is now often considered a hepatic manifestation of MetS, it does not belong to the current official criteria and will not be further discussed here, but the reader is advised to see recent reviews on the topic [13,73,74].

## 7. Conclusions

The current evidence indicates that apart from plasma triglyceride level, PXR activation can modulate all the components of the MetS (Table 2). In particular, there is robust evidence in humans that glucose tolerance is impaired and that blood pressure is elevated by PXR activation. As a major xenosensor, PXR is activated by various EDCs and PXR could mediate their metabolism-disrupting effect. Thus, PXR activation may contribute to the incidence of MetS. Emerging evidence in humans also indicates that circulating 4βHC regulates reverse cholesterol transport and blood pressure in beneficial ways. There is not yet any direct human evidence that PXR activation is a causative factor of obesity, hypertension, or type 2 diabetes. This may putatively be related to the fact that PXR activation and the effects of 4βHC may in certain aspects counteract each other.

## Figures and Tables

**Table 1:** Summary of studies on PXR and obesity.

Study	Model	Observed Effect on Obesity	Suggested Mechanism
Ma and Liu 2012 [20]	Male obesity-prone ARK/J mice on HFD (60%) for 7 weeks, treated with PCN or vehicle twice weekly	PCN treatment inhibited weight gain	PCN treatment enhanced thermogenesis, reduced food intake
He et al. 2013 [18]	WT and *Pxr*-KO mice on HFD (60%) for 12 weeks	*Pxr* KO inhibited weight gain	*Pxr* KO increased energy expenditure
	*Pxr*-KO mouse line crossed with *Ob/Ob* mice and fed with chow diet	*Pxr* KO did not affect weight gain but the *Pxr* KO mice had decreased fat mass	
	Alb-VP-*Pxr* mouse line crossed with *Ob/Ob* mice and fed with chow diet	Transgenic activation of PXR reduced body weight	
Spruiell et al. 2014a [17]	Male WT and *Pxr*-KO mice on HFD (45%) for 16 weeks	*Pxr* KO inhibited weight gain	
	Male *PXR*-humanized mice on HFD (45%) for 16 weeks	*PXR* humanization inhibited weight gain	Reduced food intake, higher basal serum leptin level
Spruiell et al. 2014b [21]	Female *PXR*-humanized mice on HFD (45%) for 16 weeks	*PXR* humanization increased weight gain	Suppression of protective role of estrogen
Zhao et al. 2017 [19]	Male WT and *Pxr* KO mice on HFD (45%) for 4 weeks	*Pxr* KO inhibited weight gain	Induction of FGF15 expression in *Pxr*-KO mice, reduced lipid absorption

**Table 2:** Components of the metabolic syndrome and the suggested role of PXR and the PXR–4βHC axis in mice and humans.

Components of MetS	PXR and/or 4βHC Implicated		Suggested Mechanism		Ref.
	Mouse	Human	Mouse	Human	
Abdominal obesity	+ (or −)		Effect on energy expenditure		[17,18,20]
Elevated triglycerides	± (plasma) + (liver steatosis)		Increased lipogenesis		[71,72,74]
Low HDL cholesterol		+		Obesity-repressed 4βHC	[67]
Hypertension	+	+	Vascular vasoconstrictive effects	Vascular vasoconstrictive effects?	[14,44]
Hyperglycemia	+	+	Reduced hepatic glucose uptake	Increased gluconeogenesis, reduced hepatic glucose uptake	[22,25]

+, increasing effect; −, decreasing effect; ±, no effect.

## References

[1] Mottillo S., Filion K.B., Genest J., Joseph L., Pilote L., Poirier P., Rinfret S., Schiffrin E.L., Eisenberg M.J. (2010). The metabolic syndrome and cardiovascular risk a systematic review and meta-analysis. J. Am. Coll. Cardiol..

[2] Ford E.S., Li C., Sattar N. (2008). Metabolic Syndrome and Incident Diabetes: Current state of the evidence. Diabetes Care.

[3] Moore J.X., Chaudhary N., Akinyemiju T. (2017). Metabolic Syndrome Prevalence by Race/Ethnicity and Sex in the United States, National Health and Nutrition Examination Survey, 1988–2012. Prev. Chronic Dis..

[4] Grundy S.M. (2008). Metabolic syndrome pandemic. Arterioscler. Thromb. Vasc. Biol..

[5] Alberti K.G., Eckel R.H., Grundy S.M., Zimmet P.Z., Cleeman J.I., Donato K.A., Fruchart J.C., James W.P., Loria C.M., Smith S.C. (2009). Harmonizing the metabolic syndrome: A joint interim statement of the International Diabetes Federation Task Force on Epidemiology and Prevention; National Heart, Lung, and Blood Institute; American Heart Association; World Heart Federation; International Atherosclerosis Society; and International Association for the Study of Obesity. Circulation.

[6] Heindel J.J., Blumberg B., Cave M., Machtinger R., Mantovani A., Mendez M.A., Nadal A., Palanza P., Panzica G., Sargis R. (2017). Metabolism disrupting chemicals and metabolic disorders. Reprod. Toxicol..

[7] Lind L., Lind P.M. (2012). Can persistent organic pollutants and plastic-associated chemicals cause cardiovascular disease?. J. Intern. Med..

[8] Neel B.A., Sargis R.M. (2011). The Paradox of Progress: Environmental Disruption of Metabolism and the Diabetes Epidemic. Diabetes.

[9] Hukkanen J., Hakkola J., Rysa J. (2014). Pregnane X receptor (PXR) - a contributor to the diabetes epidemic?. Drug Metabol. Drug Interact..

[10] Baillie-Hamilton P.F. (2002). Chemical Toxins: A Hypothesis to Explain the Global Obesity Epidemic. J. Altern. Complement. Med..

[11] Grün F., Blumberg B. (2009). Endocrine disrupters as obesogens. Mol. Cell. Endocrinol..

[12] Papalou O., Kandaraki E.A., Papadakis G., Diamanti-Kandarakis E. (2019). Endocrine Disrupting Chemicals: An Occult Mediator of Metabolic Disease. Front. Endocrinol..

[13] Hakkola J., Rysä J., Hukkanen J. (2016). Regulation of hepatic energy metabolism by the nuclear receptor PXR. Biochim. Biophys. Acta Bioenerg..

[14] Hassani-Nezhad-Gashti F., Salonurmi T., Hautajärvi H., Rysä J., Hakkola J., Hukkanen J. (2020). Pregnane X Receptor Activator Rifampin Increases Blood Pressure and Stimulates Plasma Renin Activity. Clin. Pharmacol. Ther..

[15] Heindel J.J., Newbold R., Schug T.T. (2015). Endocrine disruptors and obesity. Nat. Rev. Endocrinol..

[16] Casals-Casas C., Desvergne B. (2011). Endocrine Disruptors: From Endocrine to Metabolic Disruption. Annu. Rev. Physiol..

[17] Spruiell K., Richardson R.M., Cullen J.M., Awumey E.M., Gonzalez F.J., Gyamfi M.A. (2013). Role of Pregnane X Receptor in Obesity and Glucose Homeostasis in Male Mice. J. Biol. Chem..

[18] He J., Gao J., Xu M., Ren S., Stefanovic-Racic M., O’Doherty R.M., Xie W. (2013). PXR Ablation Alleviates Diet-Induced and Genetic Obesity and Insulin Resistance in Mice. Diabetes.

[19] Zhao L.-Y., Xu J.-Y., Shi Z., Englert N.A., Zhang S.-Y. (2017). Pregnane X receptor (PXR) deficiency improves high fat diet-induced obesity via induction of fibroblast growth factor 15 (FGF15) expression. Biochem. Pharmacol..

[20] Ma Y., Liu D. (2012). Activation of Pregnane X Receptor by Pregnenolone 16 α-carbonitrile Prevents High-Fat Diet-Induced Obesity in AKR/J Mice. PLoS ONE.

[21] Spruiell K., Jones D.Z., Cullen J.M., Awumey E.M., Gonzalez F.J., Gyamfi M.A. (2014). Role of human pregnane X receptor in high fat diet-induced obesity in pre-menopausal female mice. Biochem. Pharmacol..

[22] Rysä J., Buler M., Savolainen M.J., Ruskoaho H., Hakkola J., Hukkanen J. (2013). Pregnane X Receptor Agonists Impair Postprandial Glucose Tolerance. Clin. Pharmacol. Ther..

[23] Stage T.B., Damkier P., Christensen M.M.H., Nielsen L.B.-K., Højlund K., Nielsen F. (2015). Impaired Glucose Tolerance in Healthy Men Treated with St. John’s Wort. Basic Clin. Pharmacol. Toxicol..

[24] Ling Z., Shu N., Xu P., Wang F., Zhong Z., Sun B., Li F., Zhang M., Zhao K., Tang X. (2016). Involvement of pregnane X receptor in the impaired glucose utilization induced by atorvastatin in hepatocytes. Biochem. Pharmacol..

[25] Hassani-Nezhad-Gashti F., Rysä J., Kummu O., Näpänkangas J., Buler M., Karpale M., Hukkanen J., Hakkola J. (2018). Activation of nuclear receptor PXR impairs glucose tolerance and dysregulates GLUT2 expression and subcellular localization in liver. Biochem. Pharmacol..

[26] Hukkanen J., Rysa J., A Makela K., Herzig K.-H., Hakkola J., Savolainen M.J. (2015). The effect of pregnane X receptor agonists on postprandial incretin hormone secretion in rats and humans. J. Physiol. Pharmacol. Off. J. Pol. Physiol. Soc..

[27] Postic C., Shiota M., Niswender K.D., Jetton T.L., Chen Y., Moates J.M., Shelton K.D., Lindner J., Cherrington A.D., Magnuson M.A. (1999). Dual roles for glucokinase in glucose homeostasis as determined by liver and pancreatic beta cell-specific gene knock-outs using Cre recombinase. J. Biol. Chem..

[28] Burcelin R., Muñoz M.D.C., Guillam M.T., Thorens B. (2000). Liver hyperplasia and paradoxical regulation of glycogen metabolism and glucose-sensitive gene expression in GLUT2-null hepatocytes. Further evidence for the existence of a membrane-based glucose release pathway. J. Biol. Chem..

[29] Bhalla S., Ozalp C., Fang S., Xiang L., Kemper J.K. (2004). Ligand-activated Pregnane X Receptor Interferes with HNF-4 Signaling by Targeting a Common Coactivator PGC-1α. J. Biol. Chem..

[30] Kodama S., Moore R., Yamamoto Y., Negishi M. (2007). Human nuclear pregnane X receptor cross-talk with CREB to repress cAMP activation of the glucose-6-phosphatase gene. Biochem. J..

[31] Kodama S., Koike C., Negishi M., Yamamoto Y. (2004). Nuclear Receptors CAR and PXR Cross Talk with FOXO1 To Regulate Genes That Encode Drug-Metabolizing and Gluconeogenic Enzymes. Mol. Cell. Biol..

[32] Staudinger J.L., Goodwin B., Jones S.A., Hawkins-Brown D., MacKenzie K.I., Latour A., Liui Y., Klaasseni C.D., Brown K.K., Reinhard J. (2001). The nuclear receptor PXR is a lithocholic acid sensor that protects against liver toxicity. Proc. Natl. Acad. Sci. USA.

[33] Xie W., Barwick J.L., Downes M., Blumberg B., Simon C.M., Nelson M.C., Neuschwander-Tetri B.A., Brunt E.M., Guzelian P.S., Evans R.M. (2000). Humanized xenobiotic response in mice expressing nuclear receptor SXR. Nat. Cell Biol..

[34] Gotoh S., Negishi M. (2013). Serum- and glucocorticoid-regulated kinase 2 determines drug-activated pregnane X receptor to induce gluconeogenesis in human liver cells. J. Pharmacol. Exp. Ther..

[35] Gotoh S., Negishi M. (2015). Statin-activated nuclear receptor PXR promotes SGK2 dephosphorylation by scaffolding PP2C to induce hepatic gluconeogenesis. Sci. Rep..

[36] Gotoh S., Miyauchi Y., Moore R., Negishi M. (2017). Glucose elicits serine/threonine kinase VRK1 to phosphorylate nuclear pregnane X receptor as a novel hepatic gluconeogenic signal. Cell. Signal..

[37] Oladimeji P., Lin W., Brewer C.T., Chen T. (2017). Glucose-dependent regulation of pregnane X receptor is modulated by AMP-activated protein kinase. Sci. Rep..

[38] Hassani-Nezhad-Gashti F., Kummu O., Karpale M., Rysä J., Hakkola J. (2019). Nutritional status modifies pregnane X receptor regulated transcriptome. Sci. Rep..

[39] Zhai Y., Pai H.V., Zhou J., Amico J., Vollmer R.R., Xie W. (2007). Activation of Pregnane X Receptor Disrupts Glucocorticoid and Mineralocorticoid Homeostasis. Mol. Endocrinol..

[40] Watlington C.O., Kramer L.B., Schuetz E.G., Zilai J., Grogan W.M., Guzelian P., Gizek F., Schoolwerth A.C. (1992). Corticosterone 6 beta-hydroxylation correlates with blood pressure in spontaneously hypertensive rats. Am. J. Physiol. Physiol..

[41] Swales K.E., Moore R., Truss N.J., Tucker A., Warner T.D., Negishi M., Bishop-Bailey D. (2011). Pregnane X receptor regulates drug metabolism and transport in the vasculature and protects from oxidative stress. Cardiovasc. Res..

[42] Hagedorn K.A., Cooke C.-L., Falck J.R., Mitchell B.F., Davidge S.T. (2007). Regulation of Vascular Tone During Pregnancy. Hypertension.

[43] Wang X., Fang X., Zhou J., Chen Z., Zhao B., Xiao L., Liu A., Li Y.-S.J., Shyy J.Y.-J., Guan Y. (2013). Shear stress activation of nuclear receptor PXR in endothelial detoxification. Proc. Natl. Acad. Sci. USA.

[44] Pulakazhi Venu V.K., Saifeddine M., Mihara K., Tsai Y.C., Nieves K., Alston L., Mani S., McCoy K.D., Hollenberg M.D., Hirota S.A. (2019). PMC6732469; The pregnane X receptor and its microbiota-derived ligand indole 3-propionic acid regulate endothelium-dependent vasodilation. Am. J. Physiol Endocrinol Metab.

[45] Esposito G., Gigli S., Seguella L., Nobile N., D’Alessandro A., Pesce M., Capoccia E., Steardo L., Cirillo C., Cuomo R. (2016). Rifaximin, a non-absorbable antibiotic, inhibits the release of pro-angiogenic mediators in colon cancer cells through a pregnane X receptor-dependent pathway. Int. J. Oncol..

[46] Fisher J.P., Paton J.F.R. (2011). The sympathetic nervous system and blood pressure in humans: Implications for hypertension. J. Hum. Hypertens..

[47] Schweda F., Kurtz A. (2009). Regulation of Renin Release by Local and Systemic Factors. Rev. Physiol. Biochem. Pharmacol..

[48] Diczfalusy U., Nylen H., Elander P., Bertilsson L. (2011). 4beta-Hydroxycholesterol, an endogenous marker of CYP3A4/5 activity in humans. Br. J. Clin. Pharmacol..

[49] Janowski B.A., Willy P.J., Devi T.R., Falck J.R., Mangelsdorf D.J. (1996). An oxysterol signalling pathway mediated by the nuclear receptor LXRα. Nat. Cell Biol..

[50] Nury T., Samadi M., Varin A., Lopez T., Zarrouk A., Boumhras M., Riedinger J.-M., Masson D., Vejux A., Lizard G. (2013). Biological activities of the LXRα and β agonist, 4β-hydroxycholesterol, and of its isomer, 4α-hydroxycholesterol, on oligodendrocytes: Effects on cell growth and viability, oxidative and inflammatory status. Biochimie.

[51] Kuipers I., Van Der Harst P., Kuipers F., Van Genne L., Goris M., Lehtonen J.Y., Van Veldhuisen D.J., Van Gilst W.H., A De Boer R. (2010). Activation of liver X receptor-α reduces activation of the renal and cardiac renin–angiotensin–aldosterone system. Lab. Investig..

[52] Mitro N., Vargas L., Romeo R., Koder A., Saez E. (2007). T0901317 is a potent PXR ligand: Implications for the biology ascribed to LXR. FEBS Lett..

[53] Valbuena-Diez A.C., Blanco F.J., Oujo B., Langa C., Gonzalez-Nuñez M., Llano E., Pendas A.M., Díaz M., Castrillo A., Lopez-Novoa J.M. (2012). Oxysterol-Induced Soluble Endoglin Release and Its Involvement in Hypertension. Circulation.

[54] Morello F., De Boer R.A., Steffensen K.R., Gnecchi M., Chisholm J.W., Boomsma F., Anderson L.M., Lawn R.M., Gustafsson J.A., Lopez-Ilasaca M. (2005). Liver X receptors alpha and beta regulate renin expression in vivo. J. Clin Invest..

[55] Hayashi T., Kotani H., Yamaguchi T., Taguchi K., Iida M., Ina K., Maeda M., Kuzuya M., Hattori Y., Ignarro L.J. (2014). Endothelial cellular senescence is inhibited by liver X receptor activation with an additional mechanism for its atheroprotection in diabetes. Proc. Natl. Acad. Sci. USA.

[56] Bochud M., Bovet P., Burnier M., Eap C.B. (2009). CYP3A5andABCB1genes and hypertension. Pharmacogenomics.

[57] Xi B., Wang C., Liu L., Zeng T., Liang Y., Li J., Mi J. (2011). Association of the CYP3A5 polymorphism (6986G>A) with blood pressure and hypertension. Hypertens. Res..

[58] Lee S.D., Tontonoz P. (2015). Liver X receptors at the intersection of lipid metabolism and atherogenesis. Atherosclerosis.

[59] Temel R.E., Brown J.M. (2015). A new model of reverse cholesterol transport: EnTICEing strategies to stimulate intestinal cholesterol excretion. Trends Pharmacol. Sci..

[60] A Nikkila E., Kaste M., Ehnholm C., Viikari J. (1978). Increase of serum high-density lipoprotein in phenytoin users. BMJ.

[61] O’Neill B., Callaghan N., Stapleton M., Molloy W. (2009). Serum elevation of high density lipoprotein (HDL) cholesterol in epileptic patients taking carbamazepine or phenytoin. Acta Neurol. Scand..

[62] Luoma P.V., Sotaniemi E.A., Pelkonen R.O., Myllyla V.V. (1980). Plasma high-density lipoprotein cholesterol and hepatic cytochrome P-450 concentrations in epileptics undergoing anticonvulsant treatment. Scand. J. Clin. Lab. Investig..

[63] Choong E., Polari A., Kamdem R.H., Gervasoni N., Spisla C., Sirot E.J., Bickel G.G., Bondolfi G., Conus P., Eap C.B. (2013). Pharmacogenetic Study on Risperidone Long-Acting Injection. J. Clin. Psychopharmacol..

[64] Miller M., Burgan R.G., Osterlund L., Segrest J.P., Garber D.W. (1995). A Prospective, Randomized Trial of Phenytoin in Nonepileptic Subjects With Reduced HDL Cholesterol. Arter. Thromb. Vasc. Biol..

[65] Kojima H., Sata F., Takeuchi S., Sueyoshi T., Nagai T. (2011). Comparative study of human and mouse pregnane X receptor agonistic activity in 200 pesticides using in vitro reporter gene assays. Toxicology.

[66] Carlson L.A., Kolmodin-Hedman B. (2009). HYPER-α-LIPOPROTEINEMIA IN MEN EXPOSED TO CHLORINATED HYDROCARBON PESTICIDES. Acta Medica Scand..

[67] Salonurmi T., Nabil H., Ronkainen J., Hyotylainen T., Hautajarvi H., Savolainen M.J., Tolonen A., Oresic M., Kansakoski P., Rysa J. (2020). 4beta-Hydroxycholesterol Signals From the Liver to Regulate Peripheral Cholesterol Transporters. Front. Pharmacol..

[68] Zanger U.M., Schwab M. (2013). Cytochrome P450 enzymes in drug metabolism: Regulation of gene expression, enzyme activities, and impact of genetic variation. Pharmacol. Ther..

[69] Tremblay-Franco M., Zerbinati C., Pacelli A., Palmaccio G., Lubrano C., Ducheix S., Guillou H., Iuliano L. (2015). Effect of obesity and metabolic syndrome on plasma oxysterols and fatty acids in human. Steroids.

[70] Rodríguez-Morató J., Goday A., Langohr K., Pujadas M., Civit E., Pérez-Mañá C., Papaseit E., Ramon J.M., Benaiges D., Castañer O. (2019). Short- and medium-term impact of bariatric surgery on the activities of CYP2D6, CYP3A4, CYP2C9, and CYP1A2 in morbid obesity. Sci. Rep..

[71] Gwag T., Meng Z., Sui Y., Helsley R.N., Park S.-H., Wang S., Greenberg R.N., Zhou C. (2019). Non-nucleoside reverse transcriptase inhibitor efavirenz activates PXR to induce hypercholesterolemia and hepatic steatosis. J. Hepatol..

[72] Meng Z., Gwag T., Sui Y., Park S.-H., Zhou X., Zhou C. (2019). The atypical antipsychotic quetiapine induces hyperlipidemia by activating intestinal PXR signaling. JCI Insight.

[73] Staudinger J.L. (2019). Clinical applications of small molecule inhibitors of Pregnane X receptor. Mol. Cell. Endocrinol..

[74] Cave M.C., Clair H.B., Hardesty J.E., Falkner K.C., Feng W., Clark B.J., Sidey J., Shi H., Aqel B.A., McClain C.J. (2016). Nuclear receptors and nonalcoholic fatty liver disease. Biochim. Biophys. Acta Bioenerg..

